# Ovarian Torsion: Presentation and Management in a Pediatric Patient

**DOI:** 10.1155/2022/9419963

**Published:** 2022-02-22

**Authors:** Katie P. Nguyen, William L. Valentino, Duy Bui, Honey Milestone

**Affiliations:** ^1^Department of Family Medicine, Riverside Community Hospital, Graduate Medical Education, HCA Healthcare, 4445 Magnolia Ave, Riverside, CA 92501, USA; ^2^Department of Radiology, Riverside Community Hospital, Graduate Medical Education, HCA Healthcare, 4445 Magnolia Ave, Riverside, CA 92501, USA; ^3^Department of Obstetrics & Gynecology, Riverside Community Hospital, Graduate Medical Education, HCA Healthcare, 4445 Magnolia Ave, Riverside, CA 92501, USA

## Abstract

**Background:**

Adnexal torsion is the fifth most common gynecologic emergency accounting for approximately 20 to 30% of ovarian surgeries in pediatric patients.

**Case:**

The patient is a ten-year-old female who presented to the emergency room for severe left lower quadrant abdominal pain. On presentation, she was hemodynamically stable with an acute abdomen. A transabdominal ultrasound showed a predominantly anechoic structure measuring up to 5.6 cm without definitive Doppler flow, concerning for a large cyst causing ovarian torsion. Gynecology was consulted, and the patient underwent a diagnostic laparoscopy, aspiration of the left ovarian cyst, and left ovarian detorsion. Pathology results were consistent with benign cystic contents.

**Conclusion:**

Appropriate diagnosis and timely surgical gynecological intervention allowed this pediatric patient to salvage and preserve ovarian function.

## 1. Introduction

Ovarian torsion is caused by twisting of the ovary around the infundibulopelvic ligament and/or the utero-ovarian ligament resulting in partial or complete obstruction of its blood supply. A national cohort analysis estimates the incidence of ovarian torsion as approximately 4.9 per 100,000 in females aged 1 to 20 years in the United States [1]. Premenarchal patients are more likely to have torsion without an adnexal mass and are at higher risk of ovarian necrosis [2]. Presentation is usually sudden-onset nonradiating abdominal pain associated with nausea and vomiting [3]. This case reports on a postmenarchal patient who presents with ovarian torsion from a large simple ovarian cyst.

## 2. Case Presentation

**Figure 1:**
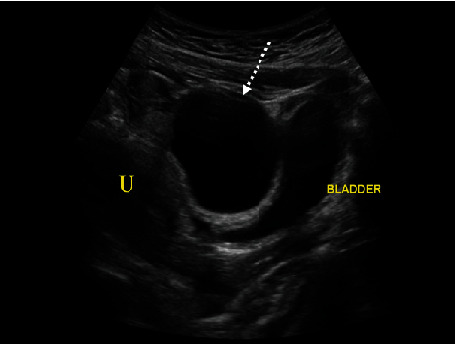
Transabdominal sagittal view of the pelvis demonstrates a large anechoic structure (dashed white arrow) located superior to the bladder and anterior to the uterus (U).

**Figure 2:**
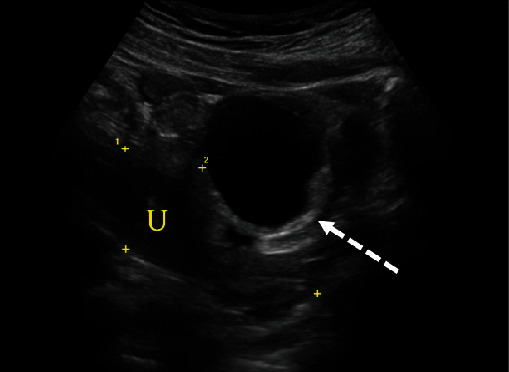
Transabdominal sagittal view demonstrates the uterus outlined in calipers (U). The view anterior to the uterus demonstrates an anechoic simple cyst. The dashed white arrow demonstrates a hyperechoic rim of displaced ovarian parenchyma with a paucity of follicles.

**Figure 3:**
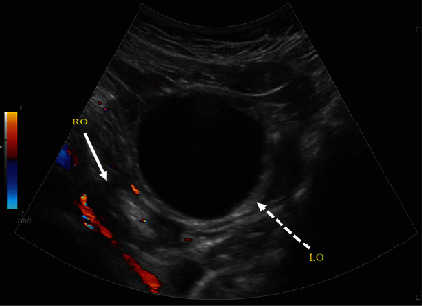
Transabdominal sagittal view of the pelvis with color Doppler, inferior to the uterus, demonstrates the right ovary (solid white arrow) with normal vascular flow. However, the left ovary (dashed white arrow), displaced by a large anechoic simple cyst, does not demonstrate vascular flow.

The patient is a ten-year-old female who presented to the emergency room for severe left lower quadrant abdominal pain. She reported pain that started ten hours prior to associated nausea. The pain was sudden in onset, constant, and 10/10 in severity without any alleviating factors. Menarche was approximately five months prior. Menstrual cycles occurred monthly, lasting five days. Her surgical history was significant for an appendectomy. She denied any fevers, diarrhea, constipation, vaginal bleeding, dysuria, or trauma. The rest of her history and labs were unremarkable. On presentation, the patient was hemodynamically stable but appeared to be in significant pain. A transabdominal ultrasound showed a large predominantly anechoic structure, i.e., simple cyst, in the midline pelvis measuring up to 5.6 cm superior to the bladder and anterior to the uterus (Figure 1). In cases of a large cystic mass, it could easily be inadvertently identified as the urinary bladder or even a bladder diverticulum. Thus, images which clearly demonstrate a cyst to be independent of the bladder are critical. The left ovary was not definitively visualized, but careful assessment of the large simple cyst demonstrated a hyperechoic rim of tissue representing a displaced left ovary (Figure 2). Doppler assessment of the large simple cyst and displaced left ovary demonstrated no discernible vascularity concerning ovarian torsion. Normal vascular flow was noted in the right ovary (Figure 3).

**Figure 4:**
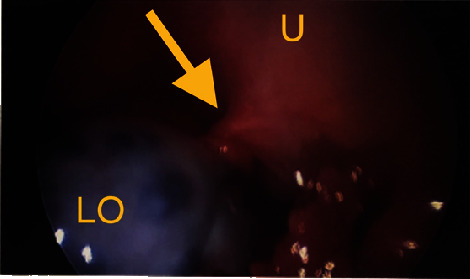
Intraoperative view of the uterus (U), left ovary (LO) with bluish hue and twisted utero-ovarian ligament (yellow arrow).

**Figure 5:**
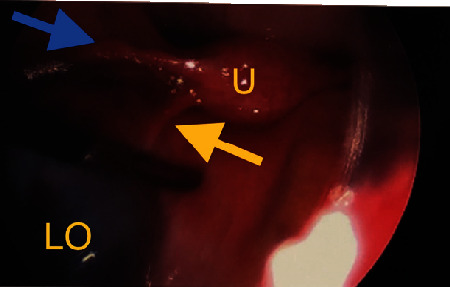
Intraoperative view of the uterus (U), round ligament (blue arrow), detorsed utero-ovarian ligament (yellow arrow) and left ovary (LO).

**Figure 6:**
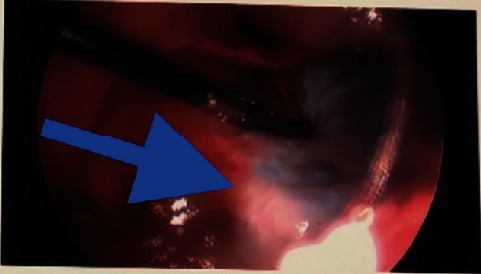
Intraoperative view showing reperfusion (blue arrow) after detorsion of infundibulopelvic and utero-ovarian ligaments.

Given the patient's acute abdomen and ultrasound findings, ovarian torsion could not be ruled out without intraoperative evaluation. Gynecology was consulted where emergent operative evaluation was discussed to preserve ovarian function. The patient's mother gave consent, and the patient underwent a diagnostic laparoscopy, left ovarian detorsion, and aspiration of the left ovarian cyst. Operative findings included a 10 cm left ovarian simple-appearing cyst with a bluish hue. Torsion was noted at the infundibulopelvic ligament and utero-ovarian ligament which were successfully detorsed (Figures 4–6). Given concerns for the inability to safely dissect the cyst wall from the ovarian cortex due to significant edema, the cyst fluid was aspirated. The patient tolerated the procedure well with an estimated blood loss of 25 ml and was transferred to recovery in stable condition. She was stable for discharge several hours postoperatively. Pathology results were negative for malignant cells and consistent with benign cystic contents of the left ovary.

## 3. Discussion

Ovarian torsion accounts for approximately 20 to 30 percent of ovarian surgeries in pediatric patients between 9 and 14 years old [4]. Adnexal torsion is the fifth most common gynecologic emergency [3]. This case reports on a pediatric patient who recently underwent menarche who presents with ovarian torsion from an ovarian cyst on the left side. The majority of adnexal torsion, i.e., 64%, occurs on the right side as the left side is protected by the descending colon [5]. The exact time needed for vascular interruption to cause irreversible damage to the ovary is unknown, but studies suggest a sharp decrease in ovarian function to be around 72 hours after symptom onset [5, 6]. This patient presented to the emergency room 10 hours after symptom onset. Symptoms are generally sudden-onset nonradiating abdominal pain which is intermittent and associated with nausea and vomiting. Premenarchal patients are more likely to present with diffuse pain instead of localized pain, fever, restlessness, palpable pelvic mass, bluish/black ovary at surgery, and longer duration of symptoms when compared to postmenarchal patients [4].

Most commonly, adolescents with adnexal torsion are found to have benign teratomas and benign functional ovarian cysts [7] as confirmed on pathology for this patient. In as many as 46% of cases, adnexal torsion in pediatric and adolescent females involves an ovary without an associated adnexal mass [8]. Premenarchal patients are thought to have elongated utero-ovarian ligaments which may increase the risk of adnexal twisting and torsion [7, 9, 10] by allowing excessive movement. The ligaments shorten with pubertal maturation. A bimanual examination is generally not necessary or tolerated in the pediatric and adolescent population. Laboratory testing is generally not useful for diagnosis of ovarian torsion as abnormalities are generally absent [7].

Diagnostic procedures may include pelvic ultrasound, color Doppler ultrasound, computed tomography, magnetic resonance imaging, endorectal ultrasound, and diagnostic laparoscopy. Pelvic ultrasound is the modality of choice when torsion is suspected particularly in premenopausal women due to the lack of ionizing radiation, and the overall diagnostic accuracy is reported to be 79% compared to 42% on CT [11]. Although there are no sufficient clinical or imaging criteria to confirm the preoperative diagnosis of adnexal torsion, patients commonly presenting with pain and the presence of a pelvic mass measuring 5 cm or larger on imaging have an 83% sensitivity for ovarian torsion [12]. The primary (and sometimes only) ultrasound finding of adnexal torsion is an enlarged ovary (53-85% of confirmed cases). The expected ovarian volume for a premenarchal child is 1-2 cm^3^. The next most common ultrasound finding is increased central echogenicity (40-85% of confirmed cases) thought to be secondary to stromal edema and/or hemorrhage [13]. Doppler studies are helpful in diagnosis when results show limited or no flow, but this should not guide clinical decision-making. Vascular flow can be normal in a torsed ovary with intermittent torsion and detorsion. Multiple ovarian follicles should be seen on ultrasound even in premenarchal patients. Concerning signs for torsion include abnormally small or paucity of follicle and peripherally displaced follicles within the ovary [3]. In this patient, the cyst is displacing the ovarian tissue with no visualized follicles and no vascular flow on Doppler studies. The whirlpool sign refers to Doppler identification of twisted blood vessels in the pedicle which is highly specific but not often visualized [14].

If findings are suggestive of adnexal torsion, surgical evaluation is promptly indicated. If findings are not suggestive of adnexal torsion, evaluate for other etiologies if the pain persists versus observation and precautions for intermittent torsion [3]. CT is not indicated for patients with suspected torsion. The imaging features of CT often demonstrate an asymmetrically enlarged ovary. However, normal CT results have a high negative predictive value when both ovaries are visualized [15]. MRI is reserved for indeterminate cases but results in delayed treatment and is therefore rarely used. Imaging features on MRI are best visualized on T2-weighted sequences without fat saturation. T1-weighted sequences with fat saturation can be useful for identifying hemorrhage [16].

A laparoscopic approach is highly recommended in an emergency situation with a remarkable clinical exam (i.e., sudden-onset severe pelvic pain and acute abdomen) to preserve ovarian function and future fertility. In 50% of cases, torsion is not found at laparoscopy [17, 18]. Minimally invasive surgery with detorsion and preservation of the adnexal structures regardless of the appearance of the ovary is recommended as the standard treatment of care for adolescents with adnexal torsion. As seen in this patient, even though intraoperative findings of a black or blue ovary suggest necrosis, this is not a reliable indicator of ovarian viability and oophorectomy is generally not necessary. Multiple studies report future ovarian function despite grossly ischemia intraoperative findings. It can take around 36 hours after detorsion to see improvement in the color of the ovary [3]. Due to significant edema and concern that dissection may further compromise vascular perfusion, aspiration of the cyst is on occasion safer than cystectomy, which was the case for this patient. If needed, a two-staged procedure is an option that would allow time for the edema to decrease and reperfusion to occur, allowing for safer dissection of the cyst wall from the ovarian cortex. Oophorectomy should be avoided unless the ovary is nonviable, malignancy is suspected, or the patient is postmenopausal. Oophorectomies are also not indicated to prevent venous thromboembolism after detorsion as there is no evidence in the literature to support this practice [1, 19]. Use of oral contraceptive pills or depot medroxyprogesterone acetate can be considered for ovulation suppression to prevent recurrent physiologic cysts [10, 20]. Although long-term follow-up fertility studies are pending, oophoropexy may be appropriate to prevent recurrence [21, 22].

Surgical approaches include adnexectomy, cystectomy, salpingectomy, oophorectomy, or detorsion with aspiration of cyst fluid or cystectomy. If a cystectomy is not performed or indicated, consideration should be made for incision and drainage of large cysts with repeat ultrasonography at 6-12 weeks [7, 23]. If a gynecologic pediatric surgeon is not available, an intraoperative consultation from a gynecologist should be obtained. Operative intervention is more likely to salvage the ovary when performed by a gynecologic surgeon [24]. Obstetrician-gynecologists are commonly consulted to manage adnexal torsion in adolescents with technical adaptations and specific challenges including special care for placement of ports, lower insufflation pressure, and possible multispecialty collaboration [3].

Ovarian torsion is an emergency which requires timely intervention with operative evaluation to preserve ovarian function and future fertility. Conservative management should be strongly considered when there is no underlying ovarian pathology. This present case report shows that a swift diagnosis of ovarian torsion and timely surgical intervention can salvage and preserve ovarian function.

## Data Availability

The data that support the conclusions of the study are available from the corresponding authors upon reasonable request.

## References

[1] Guthrie B. D., Adler M. D., Powell E. C. (2010). Incidence and trends of pediatric ovarian torsion hospitalizations in the United States, 2000-2006. *Pediatrics*.

[2] Prieto J. M., Kling K. M., Ignacio R. C. (2019). Premenarchal patients present differently: a twist on the typical patient presenting with ovarian torsion. *Journal of Pediatric Surgery*.

[3] ACOG Clinical Document (published from August 2019 through present): Adnexal torsion in adolescents (2019). Adnexal Torsion in Adolescents. *Obstetrics and Gynecology*.

[4] Ashwal E., Hiersch L., Krissi H. (2015). Characteristics and management of ovarian torsion in premenarchal compared with postmenarchal patients. *Obstetrics and Gynecology*.

[5] Rossi B. V., Ference E. H., Zurakowski D. (2012). The clinical presentation and surgical management of adnexal torsion in the pediatric and adolescent population. *Journal of Pediatric and Adolescent Gynecology*.

[6] Hubner N., Langer J. C., Kives S., Allen L. M. (2017). Evolution in the management of pediatric and adolescent ovarian torsion as a result of quality improvement measures. *Journal of Pediatric and Adolescent Gynecology*.

[7] Kives S., Gascon S., Dubuc É., Van Eyk N. (2017). No. 341-Diagnosis and Management of Adnexal Torsion in Children, Adolescents, and Adults. *Journal of Obstetrics and Gynaecology Canada*.

[8] Sasaki K. J., Miller C. E. (2014). Adnexal torsion: review of the literature. *Journal of Minimally Invasive Gynecology*.

[9] Germain M., Rarick T., Robins E. (1996). Management of intermittent ovarian torsion by laparoscopic oophoropexy. *Obstetrics and Gynecology*.

[10] Adeyemi-Fowode O., McCracken K. A., Todd N. J. (2018). Adnexal torsion. *Journal of Pediatric and Adolescent Gynecology*.

[11] Rey-Bellet Gasser C., Gehri M., Joseph J. M., Pauchard J. Y. (2016). Is it ovarian torsion? A systematic literature review and evaluation of prediction signs. *Pediatric Emergency Care*.

[12] Oltmann S. C., Fischer A., Barber R., Huang R., Hicks B., Garcia N. (2009). Cannot exclude torsion--a 15-year review. *Journal of Pediatric Surgery*.

[13] Dawood M. T., Naik M., Bharwani N., Sudderuddin S. A., Rockall A. G., Stewart V. R. (2021). Adnexal torsion: review of radiologic appearances. *Radiographics*.

[14] Hertzberg B. S., Middleton W. D. (2016). Ovarian torsion. *Ultrasound the Requisites*.

[15] Chang H. C., Bhatt S., Dogra V. S. (2008). Pearls and pitfalls in diagnosis of ovarian torsion. *Radiographics*.

[16] Sintim-Damoa A., Majmudar A. S., Cohen H. L., Parvey L. S. (2017). Pediatric ovarian torsion: spectrum of imaging findings. *Radiographics*.

[17] Cohen S. B., Weisz B., Seidman D. S., Mashiach S., Lidor A. L., Goldenberg M. (2001). Accuracy of the preoperative diagnosis in 100 emergency laparoscopies performed due to acute abdomen in nonpregnant women. *The Journal of the American Association of Gynecologic Laparoscopists*.

[18] Melcer Y., Maymon R., Pekar-Zlotin M., Pansky M., Smorgick N. (2018). Clinical and sonographic predictors of adnexal torsion in pediatric and adolescent patients. *Journal of Pediatric Surgery*.

[19] Dasgupta R., Renaud E., Goldin A. B. (2018). Ovarian torsion in pediatric and adolescent patients: a systematic review. *Journal of Pediatric Surgery*.

[20] Oelsner G., Shashar D. (2006). Adnexal torsion. *Clinical Obstetrics and Gynecology*.

[21] Fuchs N., Smorgick N., Tovbin Y. (2010). Oophoropexy to prevent adnexal torsion: how, when, and for whom?. *Journal of Minimally Invasive Gynecology*.

[22] Comeau I. M., Hubner N., Kives S. L., Allen L. M. (2017). Rates and Technique for Oophoropexy in Pediatric Ovarian Torsion: A Single- Institution Case Series. *Journal of Pediatric and Adolescent Gynecology*.

[23] Aziz D., Davis V., Allen L., Langer J. C. (2004). Ovarian torsion in children: is oophorectomy necessary?. *Journal of Pediatric Surgery*.

[24] Bristow R. E., Nugent A. C., Zahurak M. L., Khouzhami V., Fox H. E. (2006). Impact of surgeon specialty on ovarian-conserving surgery in young females with an adnexal mass. *The Journal of Adolescent Health*.

